# HSPB4/CRYAA Protect Photoreceptors during Retinal Detachment in Part through FAIM2 Regulation

**DOI:** 10.3390/neurolint16050068

**Published:** 2024-08-26

**Authors:** Cagri G. Besirli, Madhu Nath, Jingyu Yao, Mercy Pawar, Angela M. Myers, David Zacks, Patrice E. Fort

**Affiliations:** 1 Department of Ophthalmology and Visual Sciences, W.K. Kellogg Eye Center, University of Michigan, Ann Arbor, MI 48105, USA; cbesirli@med.umich.edu (C.G.B.);; 2 Department of Molecular and Integrative Physiology, University of Michigan, Ann Arbor, MI 48105, USA

**Keywords:** α-crystallin, Fas-apoptotic inhibitory molecule 2, Fas-mediated apoptosis, retinal detachment, photoreceptors

## Abstract

Our previous study discussed crystallin family induction in an experimental rat model of retinal detachment. Therefore, we attempted to evaluate the role of α-crystallin in photoreceptor survival in an experimental model of retinal detachment, as well as its association with the intrinsically neuroprotective protein Fas-apoptotic inhibitory molecule 2 (FAIM2). Separation of retina and RPE was induced in rat and mouse eyes by subretinal injection of hyaluronic acid. Retinas were subsequently analyzed for the presence αA-crystallin (HSPB4) and αB-crystallin (HSPB5) proteins using immunohistochemistry and immunoblotting. Photoreceptor death was analyzed using terminal deoxynucleotidyl transferase-mediated deoxyuridine triphosphate nick end labeling (TUNEL) staining and cell counts. The 661W cells subjected to FasL were used as a cell model of photoreceptor degeneration to assess the mechanisms of the protective effect of αA-crystallin and its dependence on its phosphorylation on T148. We further evaluated the interaction between FAIM2 and αA-crystallin using a co-immunoprecipitation assay. Our results showed that α-crystallin protein levels were rapidly induced in response to retinal detachment, with αA-crystallin playing a particularly important role in protecting photoreceptors during retinal detachment. Our data also show that the photoreceptor intrinsically neuroprotective protein FAIM2 is induced and interacts with α-crystallins following retinal detachment. Mechanistically, our work also demonstrated that the phosphorylation of αA-crystallin is important for the interaction of αA-crystallin with FAIM2 and their neuroprotective effect. Thus, αA-crystallin is involved in the regulation of photoreceptor survival during retinal detachment, playing a key role in the stabilization of FAIM2, serving as an important modulator of photoreceptor cell survival under chronic stress conditions.

## 1. Introduction

Retinal detachment (RD) is a common form of retinal injury, defined as separation of the neurosensory retina from the underlying retinal pigment epithelium (RPE). RD is a characteristic component of many of the major retinal diseases, including diabetic retinopathy and high myopia [1]. Retina–RPE separation results in the activation of death pathways, including the recently demonstrated key Fas pro-apoptotic pathway, within the light-sensing cells of the retina: the photoreceptors [2,3]. The effect of these pro-death pathways can be, at least temporarily, counter-balanced by the activation of pro-survival pathways including autophagy [4], interleukin-6, and hypoxia-inducible factors [5,6].

Our previous studies have shown that RD increases Fas-apoptotic inhibitory molecule 2 (FAIM2) levels in photoreceptors in vivo. Additionally, the FAIM2 levels were found to be upregulated in photoreceptor-like 661W cells in vitro upon activation of Fas signaling [3,7]. FAIM2 is an evolutionarily conserved 35 kDa membrane protein that is predominantly expressed in neuronal cells [8]. FAIM2 notably prevents apoptosis by direct interaction with Fas upstream of Fas-associated death domain containing protein (FADD). FAIM2 expression in neuronal cells such as cerebellar granule neurons and Purkinje cells increases resistance to Fas-mediated apoptosis, oxygen–glucose deprivation, and cerebral ischemia in several experimental models of neuronal injury [8,9,10].

Studies have reported the neuroprotective function of α-crystallin in the retina in diseases such as diabetes [11] and in autoimmune uveitis [12]. Its role in RD, however, is underexplored. A recent study reported that high levels of αB-crystallin were found in the vitreous of patients with rhegmatogenous retinal detachment [13]. Crystallins have been shown to be highly upregulated in a rodent model of mechanically induced retinal tear, consistent with a potential protective response in the context of retinal injury [14]. Additionally, a study by Hamadmad et al. from 2019 reported that αA-crystallin is significantly upregulated following RD in mice and humans [15].

We previously reported that several members of the crystallin family were induced at the mRNA level in an experimental rat model of retinal detachment after 7 days [16]. However, the precise time course of this induction and the exact function of crystallins during retinal detachment remain unknown. In this study, we sought to evaluate the influence of α-crystallin on photoreceptor survival and its association with FAIM2 in an experimental model of RD.

## 2. Materials and Methods

### 2.1. Animals

Long–Evans rats and the various mice used in this study were housed under a 12 h light/dark cycle with free access to standard chow and water. The αA- and αB-crystallin knockout mice have been previously characterized and described [17,18], and they were a generous gift from Dr. Wawrousek of the National Eye Institute (NEI). All animals were checked as negative for the rd8 mutation and genotyped as previously described to avoid confounding effects of this Crb1 gene mutation associated with photoreceptor defects and retinal degeneration [17,18]. All experiments were performed in accordance with the Association for Research in Vision and Ophthalmology (ARVO) Statement for the Use of Animals in Ophthalmic and Vision Research, while the animal protocol was previously verified and approved by the University Committee on Use and Care of Animals of the University of Michigan.

### 2.2. Experimental Model of Retinal Detachment

Retinal detachments were created per the protocol previously used and described in [3]. Briefly, the rats and mice were anesthetized with a mix of ketamine (100 mg/mL) and xylazine (20 mg/mL) prior to dilation of the pupils with topical phenylephrine (2.5%) and tropicamide (1%). A sclerotomy was made with a 25-gauge needle approximately 1–2 mm posterior to the limbus, paying particular attention to avoid damaging the lens. Subsequently, a subretinal needle was introduced through the sclerotomy into the vitreous cavity and then through a peripheral retinotomy into the subretinal space. The neurosensory retina was detached from the RPE by slowly injecting sodium hyaluronate (10 mg/mL; Abbott Medical Optics, Abbott Park, IL, USA). The injection was performed until approximately one-third to one-half of the neurosensory retina was detached in the left eye. Except for the introduction of the subretinal injector and injection of the sodium hyaluronate, the right eye was subjected to the same procedure and served as the internal control.

### 2.3. Immunoblot

Retinas were dissected from the RPE-choroid at various time points and prepared by homogenization in RIPA lysis buffer as previously described [19]. Protein concentrations were assessed with the Pierce BCA reagent in order to adjust all samples for equal protein amount. Retinal lysates were analyzed using the following antibodies: αA-crystallin (Santa Cruz, Dallas, TX, USA, Cat No: sc-28306) and αB-crystallin (Enzo life Sciences, Farmingdale, NY, USA, Cat No: ENZ-ABS706) and FAIM2 (Origene, Rockville, MD, USA, Cat No: TA-317786) in 1:1000 in TBST. Immunoblots were performed as previously described [19] but by using NuPage gels 4–12% and MES buffer, following the manufacturer’s instructions. The results were normalized by probing the same membrane with an antibody against ß-actin (Millipore-Sigma, St-Louis, MO, USA) as the loading control.

### 2.4. Immunofluorescence

Immunostaining was performed as previously described [3]. Briefly, whole eyes were recovered at various time points after retinal detachment and fixed in 4% paraformaldehyde overnight at 4 °C. The whole globes were then placed in a tissue processor (Tissue-Tek II; Sakura, Tokyo, Japan) for standard paraffin embedding. A standard paraffin microtome was then used to obtain 10 μm thick sections. Immunohistochemistry was then carried out starting with epitope unmasking using Proteinase K Antigen Retrieval. The sections were stained with the same rabbit polyclonal antibodies for FAIM2, αA-crystallin, or αB-crystallin and listed for immunoblotting, followed by staining with a secondary goat anti-rabbit IgG antibody coupled to Alexa fluorophore 488 and 594 (Jackson Immuno Research, West Grove, PA, USA). Cell nuclei were counterstained using Hoescht. No specific staining could be detected in the negative controls prepared by omitting the primary antibody during the incubation.

### 2.5. Cell Death Quantification

Apoptosis was measured by DNA Fragmentation ELISA (Roche Diagnostics, Indianapolis, IN, USA) in retinal lysates and terminal transferase dUTP nick end labeling (TUNEL) on retinal cross-sections following a previously described protocol [20,21]. For DNA fragmentation ELISA, retinal tissues were mechanically homogenized in lysis buffer, placed in a rocking incubator for 30 min, and then centrifuged at room temperature for 10 min at 1000× *g*. The supernatant was then moved to the ELISA plate, along with the positive and negative controls (20 µL each), before adding the immunoreagent complex. Incubation and washes were performed per the manufacturer’s instructions and followed by the addition of the colorimetric solution. Once the colorimetric reaction developed, the stop solution was added, and signal intensity was measured using a 96-well plate reader with excitation at 405 and normalized to the reference wavelength of 490 nm (SpectraMax Gemini EM; Molecular Devices, San Jose, CA, USA). TUNEL signal was detected with horseradish peroxidase on retinal cross-sections, and TUNEL-positive cell counts were expressed per surface area relative to the extent of detachment. For the cell culture experiments, cell death was assessed by counting the number of pyknotic cells in each condition (3 independent fields on 3 coverslips per condition in 3 independent experiments) and expressed as a function of the total number of cells in each field. The numbers provided are the averages of all fields counted.

### 2.6. Retinal Thickness Measurements

Retinal images were obtained using a Leica DM6000 microscope (Leica Microsystems, Wetzlar, Germany). Three nonoverlapping high-power fields (40×) of the retinal detachment were selected per section and averaged. One representative section was used per eye. The total thickness of the retina (measured from the outer edge of the ONL to the inner limiting membrane) and the ONL thickness (outer edge of the ONL to outer edge of the OPL) were measured in three places in each of three nonoverlapping high-power fields (40×) within the retinal detachment per section and averaged for each eye. Photoreceptor inner and outer segments were not included in the total retinal thickness measurement, given the variable retraction of these elements after detachment of the neurosensory retina, which does not necessarily correlate with viability of the photoreceptors after reattachment. For each experimental group, measurements were made on three sections of a minimum of 8 eyes, with each eye being from a separate animal.

### 2.7. Cell Culture

All cell experiments were repeated 3 times, each time including 3 individual technical replicates. The 661W photoreceptor cell line was generously gifted to us by Dr. Muayyad al-Ubaidi (Department of Cell Biology, University of Oklahoma Health Sciences Center, Oklahoma City, OK, USA). The 661 W cell line was maintained as previously described [3] in Dulbecco’s modified Eagle’s medium containing 10% fetal bovine serum, 300 mg/L glutamine, 32 mg/L putrescine, 40 μL/L of β-mercaptoethanol, 40 μg/L of both hydrocortisone 21-hemisuccinate and progesterone, penicillin (90 units/mL), and streptomycin (0.09 mg/mL). The cells were grown at 37 °C in a humidified atmosphere of 5% CO_2_ and 95% air.

### 2.8. Transfection

The cells were transfected with either empty vector, wild-type, phosphomimetic (T148D), or non-phosphorylatable (T148A) forms of αA-crystallin using the Neon Transfection System (Invitrogen, Waltham, MA, USA) as previously described [11]. After being trypsinized and washed in PBS, the cells were resuspended in suspension buffer and electroporated with 2.5 μg of the appropriate plasmid. The cells were then plated in 100 mm dishes before proceeding to further experimental procedures.

### 2.9. Fas Receptor Activation

The cells were treated with Fas ligand (FasL) as previously described [3]. Specifically, the cells were incubated with 500 ng/mL FasL (Recombinant Mouse Fas Ligand/TNFSF6 Protein Cat#: 6128-SA-025R&D Systems Inc., Minneapolis, MN, USA) and 250 ng/mL HA (Hemagglutinin/HA Peptide Antibody Cat. #: MAB060 R&D Systems Inc., Minneapolis, MN, USA) for 8 h. At the end of incubation, the cells were harvested and prepared as described below for immunoprecipitation analysis.

### 2.10. Immunoprecipitation

Next, 50 μL of Dynabeads protein G suspension (Dynabeads Protein G Immunoprecipitation Kit, 10007D, Life Technologies Corporation, Waltham, MA, USA) previously preincubated with FAIM2 antibody (OriGene) for 1 h at room temperature was added to 500 μg of retinal or cell culture lysates and incubated at 4 °C overnight. Following incubation, the beads were collected with a magnetic device and the bead–antibody–antigen complex was washed with immunoprecipitation buffer as previously described [3]. After the washes, the antibody–antigen complex was eluted with 30 μL of premixed Nupage LDS sample buffer and elution buffer and analyzed by immunoblotting as described above.

### 2.11. Statistical Analysis and ARRIVE Guidelines

Per the editorial policy, the described study is reported in accordance with ARRIVE guidelines (https://arriveguidelines.org, accessed on 1 February 2023). Power analysis was performed to calculate the number of animals necessary based on previous studies with the retinal detachment model and other studies with alpha-crystallin knockout animals, suggesting that groups of 6 were expected to be sufficient for expression and groups of 8 were expected to be sufficient for cell death effects. Exclusion criteria were established a priori and only included ocular infection and retinal tear post-retinal detachment procedure. Investigators could not be blinded to the mouse strain at the time of retinal detachment induction due to the lens aspect. However, at the time of post-processing, the samples were coded, and the technician performing the analysis was blinded as to the genotype or condition. Data were normalized to the corresponding ß-actin signal for all immunoblot experiments before statistical analysis. ANOVA models with heterogeneous variances, adjusted for the replication of the experiment, were fit to the data to assess differences between detached and attached retinas from the different genotypes using Prism software (Graphpad Prism, version 10.2.1, Boston, MA, USA). Reported in the figures are the means ± SEM (Standard Error of the Mean) and statistically significant differences. All analyses of in vivo and cell culture data were performed using a non-repeated measures ANOVA, followed by the SNK test for multiple comparisons.

## 3. Results

### 3.1. α-Crystallins Are Induced in the Detached Retina

**Figure 1:**
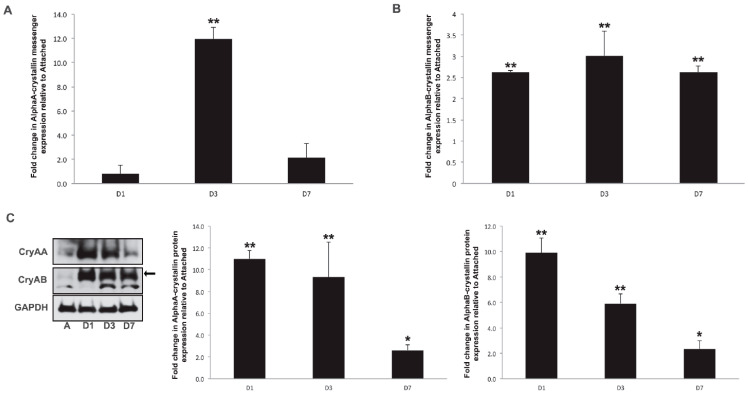
Retinal detachment is associated with differential expression of αA- and αB-crystallin. αA- (**A**) and αB-crystallin (**B**) transcripts levels were analyzed by quantitative PCR in detached and contralateral attached retinas at 1, 3, and 7 days post-detachment (*n* = 6/gp/time point). αA- and αB-crystallin protein levels (**C**) were then analyzed by immunoblotting in similar tissues, and a representative image is shown, along with a graphic representation of the corresponding quantification (the arrow denotes the specific band of interest for CryAB). While both αA- and αB-crystallin were regulated post-transcriptionally from within 1 day of retinal detachment, only αA-crystallin transcriptional expression was increased by retinal detachment transiently at the 3-day time point. * significantly different from attached (* *p* ≤ 0.05 or ** *p* ≤ 0.01). CryAA: αA- crystallin; CryAB: αB-crystallin; GAPDH: glyceraldehyde 3-phosphate dehydrogenase; A: attached; D1: day 1; D2: day 2; D7: day 7.

Retinal detachment was induced in Long–Evans rats, and its impact on α-crystallin expression was assessed to obtain a time course of expression at both the messenger RNA and protein levels. αA-crystallin messenger RNA levels showed a delayed and transient induction, peaking at 3 days post-detachment and almost shifting back to normal after 7 days (Figure 1A), while αB-crystallin messenger demonstrated a rapid induction, being detectable as early as 1 day post-detachment and consistent all the way up to 7 days post-detachment, the latest time point we analyzed (Figure 1B). We further analyzed the protein expression levels of α-crystallins in response to retinal detachment. As previously shown [11], αA- and αB-crystallins are basally expressed in the retina at very low levels. However, their levels increased significantly from 1 day after retinal detachment (Figure 1C). While less pronounced, this increase in protein level remained significant up to 7 days post-detachment, the latest time-point analyzed in our study. αB-crystallin protein levels also increased in detached retinas. Similarly to αA-crystallin, this increase could be detected after a few hours, with a peak after 1 day, and started to resolve after 7 days of retinal detachment.

**Figure 2:**
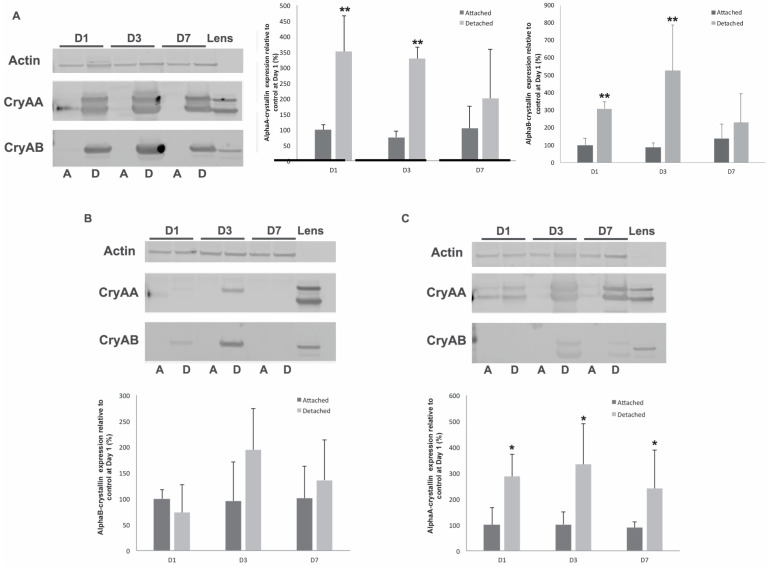
Loss of αA-crystallin affects αB-crystallin levels in response to retinal detachment, but the opposite is not true. αA- and αB-crystallin protein levels were analyzed by immunoblotting in retinas from wild-type (WT), αA-crystallin KO, and αB-crystallin KO mice at 1, 3, and 7 days post-detachment (*n* = 6/gp/time point). A representative image is shown for the WT (**A**), αA-crystallin knockout (**B**), and αB-crystallin knockout (**C**) mice, along with a graphic representation of the corresponding quantification. Both αA- and αB-crystallin protein levels were increased in WT mice. αA-crystallin knockout displayed no significant increase in αB-crystallin expression, while increased basal expression of αA-crystallin, as well as a detachment-induced increase, was observed in αB-crystallin knockout mice. * significantly different from attached (* *p* ≤ 0.05 or ** *p* ≤ 0.01). CryAA: αA- crystallin; CryAB: αB-crystallin; WT: wild type; GAPDH: glyceraldehyde 3-phosphate dehydrogenase; A: attached; D: detached; D1: day 1; D2: day 2; D7: day 7.

To assess the implications of this upregulation of α-crystallins during retinal detachment, we created detachments in wild-type, αA- and αB-crystallin knockout mice. We first analyzed the impact of retinal detachment on the expression of αA- and αB-crystallin in mice and showed that, like the rat model, αA- and αB-crystallin are rapidly elevated in response to retinal detachment, with the increase being at its peak 3 days post-detachment (Figure 2A). As expected, no αA-crystallin could be detected in the αA-crystallin knockout (Figure 2B), with only a faint signal being detected at day 3, corresponding to the known cross-reactivity of the αA-crystallin antibody with αB-crystallin. Similarly, no αB-crystallin could be detected in the αB-crystallin knockout mice (Figure 2C), with the only signal being due to some slight cross-reactivity with αA-crystallin, which was easily identifiable due to the difference in the pattern of migration and the respective signals seen in the controls (double band vs. single band at different levels). Interestingly, αB-crystallin induction was reduced in intensity and only detectable 3 days post-detachment in αA-crystallin-deficient mice when compared to the wild-type animals (Figure 2B). Conversely, the αB-crystallin knockout showed a sustained increased expression of αA-crystallin in response to retinal detachment, in addition to the previously described basal induction in this strain, which is visible in the attached retina (Figure 2C) [11]. Of note, as previously reported in several models of retinal degeneration, while the induction of alpha-crystallin in response to stress is highly reproducible, there is wide inter-individual variability for this induction, ranging from 1.5- to 50-fold.

### 3.2. Loss of αA-Crystallins, but Not αB-Crystallins, Leads to Increased Photoreceptor Death after Retinal Detachment

**Figure 3:**
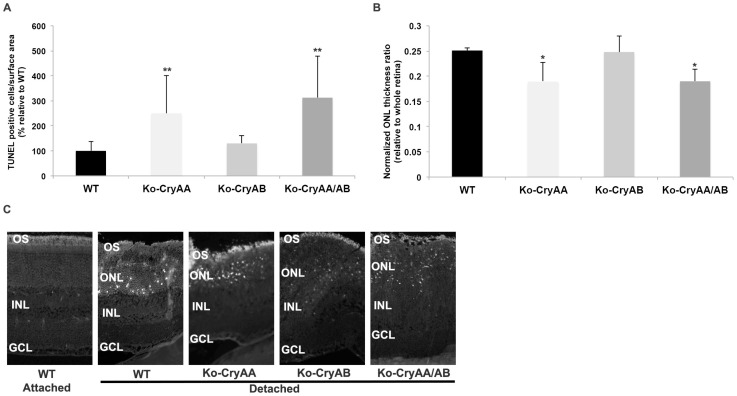
Loss of αA-crystallin, but not αB-crystallin, leads to enhanced detachment-induced photoreceptor cell death and retinal thinning. Retinal cell death (**A**) and retinal thickness (**B**) were measured on retinal cross-sections of WT mice, αA-crystallin KO mice (Ko-CryAA), αB-crystallin KO mice (Ko-CryAB), and αA/αB-crystallin double-knockout mice (Ko-CryAA/AB) at 3 days and 2 months post-retinal detachment (*n* = 9/gp/time point). The number of TUNEL-positive cells was significantly increased in the detached retinas of the Ko-CryAA and Ko-CryAA/AB mice but not in those of the Ko-CryAB mice. Representative images of TUNEL staining used for cell counts are shown in (**C**). Consistent with the observed levels of cell death, retinal thickness, especially that of ONL, was reduced in the Ko-CryAA and Ko-CryAA/AB mice but not in the Ko-CryAB mice. * significantly different from WT mice (* *p* ≤ 0.05 or ** *p* ≤ 0.01). CryAA: αA-crystallin; CryAB: αB-crystallin; WT: wild type). OS: outer segments; ONL: outer nuclear layer; INL: inner nuclear layer; GCL: ganglion cell layer.

We further analyzed the impact of retinal detachment on photoreceptor cell survival by measuring the density of TUNEL-positive cells at 3 days post-detachment, when cell death is maximal, or by measuring the thickness of the outer nuclear layer 2 months post-RD. In both cases, our data strongly support a protective role for αA-crystallin, as demonstrated by an increased number of TUNEL-positive cells (Figure 3A) and a further reduction in ONL thickness (Figure 3B) in αA-crystallin-deficient mice when compared to age-matched wild-type animals (28 in WT vs. 66 in Ko-CryAA TUNEL-positive cells per 500 µm of detached area). Consistent with previous findings in another model of retinal degeneration [11], lack of αB-crystallin, which is associated with a basal increase in αA-crystallin, did not significantly change the rate of retinal detachment-induced photoreceptor degeneration (vs. 35 in Ko-CryAB TUNEL-positive cells), consistent with a protective role for αA-crystallin. The αA- and αB-crystallin double-knockout mice demonstrated increased photoreceptor loss after retinal detachment compared to the WT controls (vs. 85 in Ko-CryAA/AB TUNEL-positive cells), similar to the phenotype seen in the αA-crystallin knockout mice (see Figure 3C for representative images).

### 3.3. α-Crystallins Are Induced and Accumulate in the Photoreceptor Inner and Outer Segments in the Detached Retina

**Figure 4:**
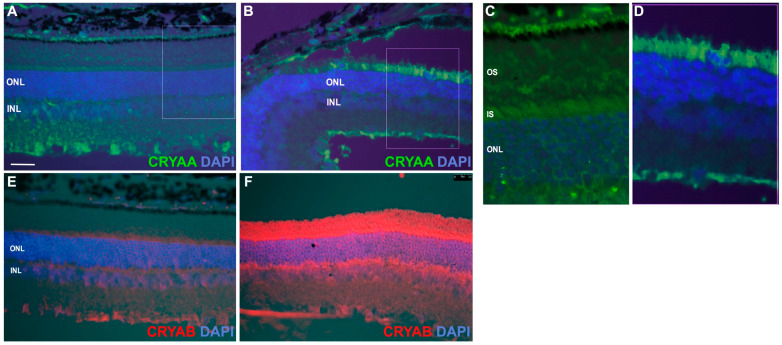
αA-crystallin and αB-crystallin are expressed in photoreceptor inner and outer segments following retinal detachment. Immunohistochemistry for αA- (**A**,**B**) and αB-crystallins (**E**,**F**) was performed on cross-sections from attached (**A**,**E**) and detached (**B**,**F**) retinas of WT mice, respectively (*n* = 3). Consistent with the protein expression data, the immunostaining results show increased immunoreactivity of both α-crystallins, specifically in photoreceptors, in response to detachment ((**C**) attached vs. (**D**) detached insert for αA-crystallin corresponding to white rectangles in A and B respectively). OS: outer segments; IS: inner segments; ONL: outer nuclear layer; INL: inner nuclear layer.

We further analyzed the cellular localization of αA- and αB-crystallin by immunofluorescence analysis on retinal cross-sections. As previously reported [11], both αA- and αB-crystallins are mainly expressed at low levels in the ganglion cell layer in the attached retina (Figure 4A). Analysis of the retina 24 h post-detachment showed that αA-crystallin staining was predominantly increased in the outer retina (Figure 4B), especially so in the inner segments of photoreceptors in the detached area, as shown in the higher-magnification images of attached (Figure 4C) and detached regions (Figure 4D). αB-crystallin was also induced in the outer retina, throughout the photoreceptor cells, from the outer segments, all the way to the outer plexiform layer (Figure 4E,F).

### 3.4. FAIM2 Expression in Stressed Photoreceptors Requires the Presence of Both α-Crystallins

**Figure 5:**
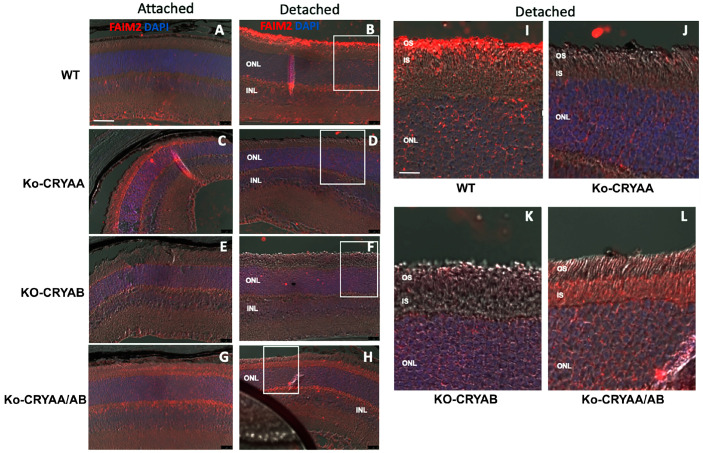
FAIM2 expression in stressed photoreceptors requires the presence of α-crystallins. Immunohistochemistry for FAIM2 was performed on cross-sections from attached (**A**,**C**,**E**,**G**) and detached (**B**,**D**,**F**,**H**) retinas of WT (**A**,**B**), αA-crystallin KO (**C**,**D**), αB-crystallin KO (**E**,**F**), and αA/αB-crystallin double-knockout (**G**,**H**) mice (*n* = 3/gp). As shown in the inserts (**I**–**L** corresponding to white rectangles in **B**, **D**, **F** and **H**), FAIM2 immunoreactivity was significantly increased in photoreceptor segments following detachment only in the WT mice. OS: outer segments; IS: inner segments; ONL: outer nuclear layer; INL: inner nuclear layer.

We recently showed that FAIM2 is an important neuroprotective factor in photoreceptors during periods of retina–RPE separation [3]. To determine the potential role of crystallins in the regulation of FAIM2 expression in the retina, we assessed cellular localization of FAIM2 in WT and α-crystallin knockout mice. This analysis confirmed increased immunoreactivity (Figure 5A,B) in the photoreceptor outer segments following retinal detachment in the WT animals. In contrast, no changes in FAIM2 levels were detected in single αA-crystallin (Figure 5C,D) or αB-crystallin (Figure 5E,F) or dual αA/αB-crystallin knockout (Figure 5G,H) retinas following retinal detachment (Figure 5D, Figure 5F, and Figure 5H, respectively). This specific induction of FAIM2 in WT was further examined and represented using higher-magnification images of the detached regions of each genotype (Figure 5I,L). These findings strongly suggest that increased FAIM2 protein in photoreceptors after retinal detachment requires the presence of both αA- and αB-crystallins.

### 3.5. Increased Interaction of FAIM2 with αA-Crystallin after Retinal Detachment

**Figure 6:**
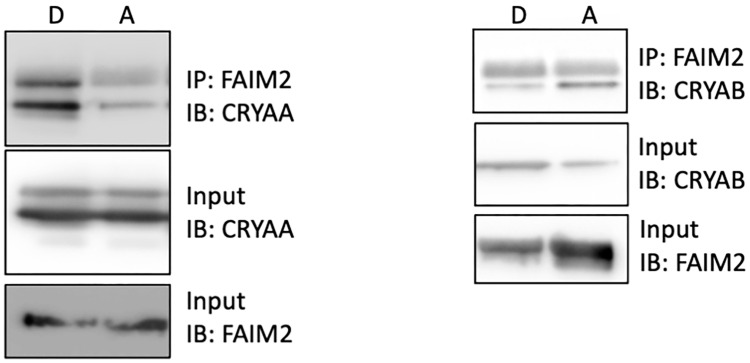
FAIM2 interacts strongly with αA-crystallin. Representative immunoblots for FAIM2 and αA- (**left panel**) and αB-crystallin (**right panel**) following co-immunoprecipitation using tissue extracts from attached and detached rat retinas (*n* = 6). Increased levels of αA-, but not αB-crystallin, were pulled-down with FAIM2 in detached retinas compared to attached retinas.

To confirm the role of αA- and αB-crystallins in the regulation of neuroprotective factors, we immunoprecipitated FAIM2 and assessed the pull-down of αA- or αB-crystallin shortly after experimental retinal detachment. We first demonstrated that both αA- and αB-crystallin showed some interaction with FAIM2 in the attached retina (Figure 6–A lanes). However, while a clear increased association of FAIM2 with αA-crystallin was detected (Figure 6–left panel), there was a decrease in FAIM2’s association with αB-crystallin in response to experimental retinal detachment (Figure 6–right panel).

### 3.6. FAIM2 Interaction with αA-Crystallin in Photoreceptors during Fas Activation Is T148 Phosphorylation-Dependent

**Figure 7:**
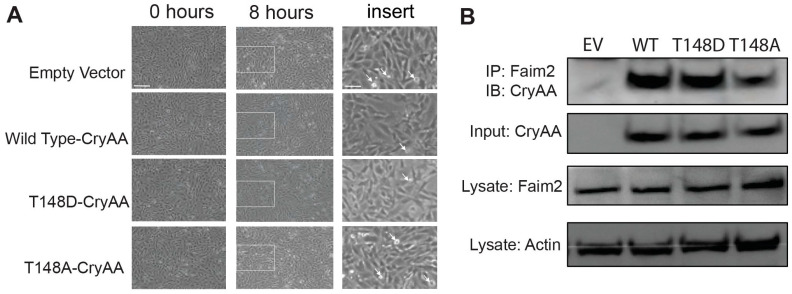
FAIM2’s interaction with αA-crystallin is T148 phosphorylation-dependent. (**A**) Representative images of 661W cells before and after 8 h of Fas pathway activation (*n* = 3 independent experiments with three replicates each). Higher-magnification (insert corresponding to the white rectangles on 8 h full fields) images are provided to better appreciate the larger number of pyknotic cells (white arrows denote some examples) in the empty vector- and T148A-CryAA-overexpressing cells compared to the wild-type and T148D-CryAA-overexpressing 661W cells. (**B**) Representative immunoblots (membranes were cut prior to hybridization for improved signal/noise) for FAIM2 and αA-crystallin following co-immunoprecipitation using cell lysates from Fas-activated photoreceptors overexpressing either the wild-type, phosphomimetic (T148D) or non-phosphorylatable (T148A) form of αA-crystallin. Increased levels of the phosphomimetic form (T148D) were pulled-down with FAIM2 in comparison to the non-phosphorylatable (T148A) form of αA-crystallin. (IP: immunoprecipitation; IB: immunoblot; EV: empty vector; WT: wild type; scale bars are 25 μm (large field) and 10 μm (insert).).

Photoreceptor death following retina–RPE separation is mediated by Fas receptor signaling and the activation of apoptosis [2]. Fas ligand-induced apoptosis of 661W cone-like cells closely mirrors this in vivo finding and has been used as a robust in vitro model [3,4]. Consistent with the protective role of αA-crystallin and its phosphorylation on T148, overexpression of either the wild-type or phosphomimetic form (T148D) of αA-crystallin reduced FasL-induced cell death by more than 40% when compared to empty vector-transfected cells (Figure 7A). Counting the number of pyknotic cells showed that 8 h of FasL treatmentled to an average of 110 cells in the EV-transfected cells compared to 58, 63, and 106 in the WT cells and T148D- and T148A-transfected 661W cells, respectively. To further assess the role of αA-crystallin’s phosphorylation on residue 148 with FAIM2 interaction during Fas activation in photoreceptors, we immunoprecipitated FAIM2 and assessed its interaction with the wild-type, phosphomimetic (T148D), or non-phosphorylatable (T148A) forms of αA-crystallin in a 661W in vitro photoreceptor cell model. The data also demonstrated that phosphorylation of αA-crystallin on residue 148 led to an increased interaction with FAIM2 in Fas-activated photoreceptors, whereas pull-down of the non-phosphorylatable form was significantly reduced (Figure 7B) This observation suggests that αA-crystallin’s phosphorylation on residue 148 is important for its interaction with FAIM2, and thus, it plays a key role during Fas-mediated cell death in photoreceptors.

## 4. Discussion

In the current study, we directly tested the role of αA- and αB-crystallin in photoreceptor survival in the context of experimental retinal detachment as a model of acute retinal injury. We demonstrate that both proteins are rapidly induced in response to retinal detachment in both rat and mouse models, and αA-crystallin’s absence is associated with increased photoreceptor cell death, while loss of αB-crystallin is not, most likely due to compensation by αA-crystallin. Our data show that FAIM2, an intrinsic neuroprotective protein in photoreceptors, interacts with αA and αB-crystallins following retinal detachment. This interaction may be important for stabilizing FAIM2 and increasing total protein levels in photoreceptors, facilitating their ability to resist apoptotic stress after retinal detachment. We further analyzed the regulation of αA-crystallin’s protective function and demonstrated that the newly described phosphorylation on S/T148 is key for its protective effect during Fas activation in photoreceptors and that this, in part, may be driven by its interaction and regulation of FAIM2 function. However, it cannot be ruled out that other protective mechanisms previously implicating αA-crystallin could also be involved. This is actually even further supported by the fact that in the αB-crystallin knockout mice, αA-crystallin was able to partially compensate and offer transient protection despite reduced recruitment of FAIM2. Altogether, our data suggest that combined induction of αA-crystallin and αB-crystallin is required for the recruitment of FAIM2 and increased protection and that sole induction of αA-crystallin is able to provide protection, in part, through different mechanisms.

Our study found a marked increase in the protein level of α-crystallin in the experimental retinal detachment model. This finding is consistent with previous reports of α-crystallin upregulation in retinal degeneration mouse models [22] and in the retina of human donors with age-related macular degeneration [23,24]. Consistent with the key role of αA-crystallin in the retina, a recent study by Hamadmad et al. has reported that, post-retinal detachment, αA-crystallin displayed the greatest increase among all crystallins in mouse tissues. They further reported a significant increase in vitreous protein levels of αA-crystallin in retinal detachment patients compared to their control counterparts [15].

Following experimental retinal detachment, α-crystallins were induced and accumulated in photoreceptor inner and outer segments. This localization is consistent with another study considering later stages of retinal detachment that reported that αA-crystallin expression is highly increased compared to control values and that its immunoreactivity is found primarily in the inner and outer segments of the photoreceptors, with slightly more diffuse staining in other retinal layers [15]. This is also consistent with another disease mostly impacting the outer retina, experimentally induced uveitis, in which αA-crystallin is also highly upregulated in the retina and primarily localized in the photoreceptor inner segments [12].

The protective role of αA-crystallin reported in our study is consistent with multiple previous studies reporting that αA-crystallin upregulation plays a protective role in the suppression of photoreceptor apoptosis associated with intraocular inflammation [25], experimental autoimmune uveitis [26], and a Rpe65⁻/⁻ mouse model of Leber congenital amaurosis [27]. On a mechanistic level, we previously reported that photoreceptor apoptosis is accelerated in FAIM2 knockout mice following experimental retinal detachment. We also reported that FAIM2 is primarily involved in reducing stress-induced photoreceptor cell death, but this effect was found to be transient [3,7]. The transient nature of the effect of FAIM2 is similar to the one we recently reported relative to αA-crystallin during another chronic condition: diabetes [11]. Thus, in the current study, we decided to assess the functional relationship between FAIM2 and αA-crystallin in photoreceptors after retinal detachment. We report here that while basal level of interaction between FAIM2 and αA- and αB-crystallin was observed in the attached retina, a significant increase in FAIM2 and αA-crystallin association was detected following retinal detachment. Altogether, our data suggest that the protective effect of αA-crystallin during retinal detachment is at least partially due to the regulation of FAIM2, but considering the previously demonstrated transient nature of this effect and the multiple pathways in which αA-crystallin is involved, the involvement of these other mechanisms cannot be ruled out.

α-Crystallins are known to undergo numerous post-translation modifications (PTMs), which further affect their chaperone activity [28,29,30,31]. We recently reported that the serine/threonine 148 residue of αA-crystallin is highly phosphorylated in the retina under normal conditions. Further, we uncovered that phosphorylation on this specific residue is significantly decreased in the retina of diabetic donors with retinopathy and that this serine/threonine 148 phosphorylation essentially controls the protective role of αA-crystallin [11]. Consistent with our previous study, we have shown that this phosphorylation is also important for the interaction of αA-crystallin with FAIM2 in 661W cells.

## 5. Conclusions

In conclusion, our current study highlights a new mechanism by which αA-crystallin is involved in the regulation of photoreceptor survival during retinal detachment, playing a key role in the stabilization of FAIM2, an important modulator of photoreceptor cell survival under chronic stress conditions such as unresolved retinal detachment.

## Data Availability

The datasets used and/or analyzed during the current study are available from the corresponding author on reasonable request.
